# Natural History Study of STXBP1-Developmental and Epileptic Encephalopathy Into Adulthood

**DOI:** 10.1212/WNL.0000000000200715

**Published:** 2022-07-19

**Authors:** Hannah Stamberger, David Crosiers, Ganna Balagura, Claudia M. Bonardi, Anna Basu, Gaetano Cantalupo, Valentina Chiesa, Jakob Christensen, Bernardo Dalla Bernardina, Colin A. Ellis, Francesca Furia, Fiona Gardiner, Camille Giron, Renzo Guerrini, Karl Martin Klein, Christian Korff, Hana Krijtova, Melanie Leffner, Holger Lerche, Gaetan Lesca, David Lewis-Smith, Carla Marini, Dragan Marjanovic, Laure Mazzola, Sarah McKeown Ruggiero, Fanny Mochel, Francis Ramond, Philipp S. Reif, Aurélie Richard-Mornas, Felix Rosenow, Christian Schropp, Rhys H. Thomas, Aglaia Vignoli, Yvonne Weber, Elizabeth Palmer, Ingo Helbig, Ingrid E. Scheffer, Pasquale Striano, Rikke S. Møller, Elena Gardella, Sarah Weckhuysen

**Affiliations:** From the Applied and Translational Neurogenomics Group (H.S., S.W.), VIB Center for Molecular Neurology, University of Antwerp; Department of Neurology (H.S., D.C., S.W.), Antwerp University Hospital; Faculty of Medicine and Health Sciences (D.C., S.W.), Translational Neurosciences, Institute Born-Bunge (D.C.), and µNEURO Research Centre of Excellence (D.C., S.W.), University of Antwerp, Belgium; IRCCS Istituto Giannina Gaslini (G.B., P.S.), Genova; Department of Neurosciences, Rehabilitation, Ophthalmology, Genetics, Maternal and Child Health (G.B., P.S.), University of Genova, Italy; Department of Epilepsy Genetics (C.M.B., F.F., R.S.M., E.G.), Danish Epilepsy Centre Filadelfia, Dianalund, Denmark; Department of Woman's and Child's Health (C.M.B.), Padova University Hospital, Italy; Population Health Sciences Institute (A.B.), Newcastle University; Department of Paediatric Neurology (A.B.), Newcastle upon Tyne Hospitals NHS Foundation Trust, United Kingdom; Child Neuropsychiatry Section (G.C.), Department of Surgical Sciences, Dentistry, Gynecology and Paediatrics, University of Verona; UOC Neuropsichiatria Infantile (G.C.), Dipartimento Materno-Infantile, Azienda Ospedaliero-Universitaria Integrata, Verona; Center for Research on Epilepsies in Pediatric Age (CREP) (G.C., B.D.B.), Verona; Epilepsy Center (V.C.), ASST Santi Paolo Carlo, Milan, Italy; Department of Clinical Medicine (J.C.), Aarhus University; Department of Neurology (J.C.), Aarhus University Hospital, Denmark; Department of Neurology (C.A.E.), University of Pennsylvania Perelman School of Medicine, Philadelphia; Institute for Regional Health Services Research (F.F., R.S.M., E.G.), University of Southern Denmark, Odense; University of Melbourne, Austin Health (F.G., I.E.S.), Victoria, Australia; AP-HP (C.G.), Pitié-Salpêtrière University Hospital, Department of Neurology, Paris, France; Child Neurology Unit and Laboratories (R.G.), Neuroscience Department, Children's Hospital A. Meyer–University of Florence, Italy; Departments of Clinical Neurosciences (K.M.K.), Medical Genetics and Community Health Sciences, Hotchkiss Brain Institute & Alberta Children's Hospital Research Institute, Cumming School of Medicine, University of Calgary, Canada; Epilepsy Center Frankfurt Rhine-Main (K.M.K., P.S.R., F. Rosenow), Department of Neurology, Johann Wolfgang Goethe University; LOEWE Center for Personalized Translational Epilepsy Research (CePTER) (K.M.K., P.S.R., F. Rosenow), Goethe University Frankfurt, Frankfurt am Main, Germany; Pediatric Neurology Unit (C.K.), University Hospitals, Geneva, Switzerland; Department of Neurology (H.K.), Second Faculty of Medicine, Charles University and Motol University Hospital Prague, Czech Republic; The GOLD Service (M.L.), Waratah, New South Wales, Australia; Department of Neurology and Epileptology & Hertie Institute for Clinical Brain Research (H.L., Y.W.), University of Tubingen, Germany; Department of Medical Genetics (G.L.), Lyon University Hospital, Université de Lyon, INMG, France; Translational and Clinical Research Institute (D.L.-S., R.H.T.), Newcastle University; Department of Clinical Neurosciences (D.L.-S., R.H.T.), Newcastle Upon Tyne Hospitals NHS Foundation Trust, United Kingdom; Child Neurology and Psychiatric Unit (C.M.), G. Salesi Pediatric Hospital, United Hospitals of Ancona, Italy; Department of Adults with Handicap (D.M.), Danish Epilepsy Centre, Dianalund, Denmark; Department of Neurology (L.M.), University Hospital of St-Etienne; Team “Central Integration of Pain” (L.M.), Lyon Neuroscience Research Center, INSERM U 1028, CNRS UMR 5292, France; The Epilepsy NeuroGenetics Initiative (ENGIN) (S.M.R.), Children's Hospital of Philadelphia, PA; AP-HP (F.M.), Pitié-Salpêtrière University Hospital, Department of Genetics, Reference Centers for Adult Neurometabolic Diseases and Adult Leukodystrophies; INSERM U 1127 (F.M.), CNRS UMR 7225, Sorbonne Universités, UPMC Univ Paris 06 UMR S 1127, Paris Brain Institute, ICM; Service de Génétique (F. Ramond), Centre Hospitalier Universitaire de Saint-Etienne, France; Department of Neurology (P.S.R.), Ortenau Klinikum Offenburg Kehl, Germany; Unit of Neurophysiology and Epileptology (A.R.-M.), Hospices Civils of Lyon, France; Kinderklinik Dritter Orden (C.S.), Passau, Germany; Child Neuropsychiatry Unit (A.V.), Department of Health Sciences, ASST Santi Paolo e Carlo, San Paolo Hospital, Università Degli Studi di Milano, Italy; Department of Epileptology and Neurology (Y.W.), University of Aachen, Germany; School of Women and Children's Health (E.P.), Faculty of Medicine, UNSW; Sydney Children's Hospital Network (E.P.), Randwick, Australia; Division of Neurology (I.H.), Children's Hospital of Philadelphia, PA; and Department of Paediatrics (I.E.S.), University of Melbourne, Royal Children's Hospital, Florey and Murdoch Children's Research Institutes, Melbourne, Australia.

## Abstract

**Background and Objectives:**

Pathogenic *STXBP1* variants cause a severe early-onset developmental and epileptic encephalopathy (STXBP1-DEE). We aimed to investigate the natural history of STXBP1-DEE in adults focusing on seizure evolution, the presence of movement disorders, and the level of functional (in)dependence.

**Methods:**

In this observational study, patients with a minimum age of 18 years carrying a (likely) pathogenic *STXBP1* variant were recruited through medical genetics departments and epilepsy centers. Treating clinicians completed clinical questionnaires and performed semistructured video examinations while performing tasks from the (modified) Unified Parkinson Disease Rating Scale when possible.

**Results:**

Thirty adult patients were included for summary statistics, with video recordings available for 19 patients. The median age at last follow-up was 24 years (range 18–58 years). All patients had epilepsy, with a median onset age of 3.5 months. At last follow-up, 80% of adults had treatment-resistant seizures despite long periods of seizure freedom in 37%. Tonic-clonic, focal, and tonic seizures were most frequent in adults. Epileptic spasms, an unusual feature beyond infancy, were present in 3 adults. All individuals had developmental impairment. Periods of regression were present in 59% and did not always correlate with flare-ups in seizure activity. Eighty-seven percent had severe or profound intellectual disability, 42% had autistic features, and 65% had significant behavioral problems. Video examinations showed gait disorders in all 12 patients able to walk, including postural abnormalities with external rotation of the feet, broad-based gait, and asymmetric posture/dystonia. Tremor, present in 56%, was predominantly of the intention/action type. Stereotypies were seen in 63%. Functional outcome concerning mobility was variable ranging from independent walking (50%) to wheelchair dependence (39%). Seventy-one percent of adults were nonverbal, and all were dependent on caregivers for most activities of daily living.

**Discussion:**

STXBP1-DEE warrants continuous monitoring for seizures in adult life. Periods of regression are more frequent than previously established and can occur into adulthood. Movement disorders are often present and involve multiple systems. Although functional mobility is variable in adulthood, STXBP1-DEE frequently leads to severe cognitive impairments and a high level of functional dependence. Understanding the natural history of STXBP1-DEE is important for prognostication and will inform future therapeutic trials.

Syntaxin-binding protein 1 (STXBP1), Sec1/Munc18, encoded by the chromosome 9 located gene *STXBP1*, is a key regulator of synaptic vesicle docking and fusion through its interaction with the soluble NSF attachment protein receptors or SNARE proteins.^1-4^ Normal functioning of STXBP1 is critical for neurotransmitter release at all synapses and essential for neuronal survival.^4-6^ Pathogenic genetic variants in *STXBP1* were first identified in 2008 in patients with Ohtahara syndrome, a severe neonatal-onset developmental and epileptic encephalopathy (DEE).^7^ Subsequently, the phenotypic spectrum of STXBP1-DEE has significantly broadened including other DEEs such as West syndrome, Lennox-Gastaut syndrome, unclassified early infantile DEEs, and, less frequently, neurodevelopmental disorders with infrequent or no seizures.^8-12^

STXBP1-DEE is usually caused by heterozygous de novo variants affecting *STXBP1*. Parental mosaicism has been reported,^13^ and recently, a homozygous missense variant has been described and functionally studied.^14^ At the protein level, haploinsufficiency/loss of function is the main proposed disease mechanism. This is supported by the fact that more than half of the reported variants are protein-truncating variants or splice-site variants and smaller or larger indels or deletions.^12^ Furthermore, missense variants can lead to decreased protein expression at the synapse.^15^ However, recent studies suggest alternative molecular mechanisms such as a dominant-negative^16,17^ or gain-of-function effect of specific missense variants.^14^ To date, no obvious genotype–phenotype correlations have been identified.

STXBP1-DEE is now recognized as one of the more frequent monogenic DEEs, with a predicted incidence of 3.30–3.81 per 100,000 births.^18^ In addition to intellectual disability and epilepsy, movement disorders such as tremor and ataxia are frequent.^12,19-22^ Extrapyramidal features have been described in a few adolescents and adults with STXBP1-DEE, although information on the prevalence of this clinical symptomatology at adult age is missing.^12,23^

To date, no studies have systematically investigated the clinical presentation of STXBP1-DEE in adulthood. Such studies are important to inform prognostication and genetic counseling and to understand the natural history of the disease with the prospect of future therapeutic trials. Here, we analyzed the clinical features of 32 adult patients with disease-causing *STXBP1* variants with special focus on epileptology in different phases of life, movement disorders, and functional independence in adulthood.

## Methods

### Patient Recruitment and Genotyping

Thirty-two patients were recruited through an international network of clinicians, mostly consisting of medical geneticists and epileptologists, following patients with STXBP1-DEE. For inclusion in the study, patients had to be at least 18 years old and have a pathogenic or likely pathogenic *STXBP1* variant according to the American College of Medical Genetics and Genomics guidelines,^24^ which was (re)assessed by the use of the program VarSome (eTable 1, links.lww.com/WNL/C93).^25^ Thirteen patients were previously published (patients 7,^26^ 8,^27^ 9,^9^ 10,^9^ 12,^28^ 14,^9,29^ 15,^30^ 18,^26^ 19,^30^ 20,^12^ 24,^12^ 28,^12^ and 31^23^), and updated information with focus on clinical features at adult age was included in this study. Variants of previously unpublished patients were identified by collaborating research and diagnostic laboratories, and parental and family segregation studies were performed where possible.

### Phenotyping

Clinical data were collected using 3 clinical questionnaires completed by referring clinicians. One questionnaire focused on epileptology and development, 1 on movement disorders and extrapyramidal features, and 1 on mobility, communication, and functional independence in adulthood. This last questionnaire integrated 2 standardized scales: the Functional Mobility Scale to evaluate mobility and the Katz scale to evaluate self-reliance concerning activities of daily living.^31^ Seizure types were classified according to the International League Against Epilepsy Classification.^32^ Age at epilepsy improvement was defined as the age at which a clinically relevant decrease in seizure frequency was seen, as noted in the medical records of the treating physicians. The reported effect of antiseizure medications (ASMs) was based on the clinical impression of the treating physician. Seizure freedom was defined as the period without seizures being at least 3 times longer than the longest interseizure interval in the preceding year.^33^ Cognitive outcome was defined based on the level of adaptive functioning as proposed by the *Diagnostic and Statistical Manual of Mental Disorders, Fifth Edition* (*DSM-5*).^34^

### Video Examination

Referring clinicians were asked to video record their patients while performing tasks of the motor subscale of the modified Unified Parkinson Disease Rating Scale (MDS-UPDRS)^35^ adapted to a level feasible for their patient(s). Because of the variability in the performance level of individual patients, the different items could not be scored numerically, but videos were systematically assessed for the presence of the following features: hypomimia, bradykinesia, postural stability, gait abnormalities, tremor, ataxic features, and stereotypies. Video recordings were reviewed independently by the clinical neurologists coordinating the study (H.S., E.G., and S.W.) and an independent neurologist specialized in movement disorders (D.C.) after which a consensus was reached.

### Statistical Analyses

Clinical data on 30 of 32 patients were included for summary statistics; data of 2 patients were excluded (see Results section). As we did not have complete information on all patients, denominators in the Results section indicate the number on whom information on the clinical feature addressed was available. A χ^2^ test was used to compare variant type (missense vs other) and seizure outcome (seizure-free vs not seizure-free), presence of regression (yes vs no), cognitive outcome (moderate-severe ID vs severe-profound ID), and functional outcome concerning mobility (ambulatory vs wheelchair dependent) and communication/speech (some speech vs nonverbal); and to compare seizure outcome with cognitive outcome and functional outcome concerning mobility and speech. A Fisher exact test was used in case any of the cells had an expected count below 5. A nonparametric Mann-Whitney *U* test was used to look for a correlation between seizure-onset age and cognitive outcome and functional outcome concerning mobility and speech. Statistical analyses were performed using SPSS statistics software (IBM SPSS Statistics 28). Reported tests were performed 2-tailed with an alpha level for significance of *p* < 0.05.

### Standard Protocol Approvals, Registrations, and Patient Consents

Written informed consent for participation/publication in this study was obtained from all participants or their parents or legal guardians. Separate consent forms for the publication of pictures/videos were obtained when applicable.

### Data Availability

Deidentified data can be shared by request from any qualified investigator.

## Results

### Study Cohort and *STXBP1* Variants

Thirty-two adult patients, 17 females and 15 males, from 31 families were referred for our study. Detailed clinical information is presented in eTable 2 (links.lww.com/WNL/C93). In this cohort, 30 different *STXBP1* variants were identified (eTable 1): 15 missense variants (the p.(Glu283Lys) variant was present in 2 unrelated patients), 6 splice-site variants, 4 stop gains, 2 frameshift variants, 2 indels, and 1 deletion. Seven of 30 were known recurrent variants. In 26 of 31 (84%) families, *STXBP1* variants occurred de novo. Two sisters carried the same p.(Ile439Phe) variant that was not detected in blood-derived DNA of their parents and 2 healthy siblings, so germline mosaicism in one of the parents was suspected. For 3 other patients, variant inheritance was unknown/incomplete, although pathogenicity was considered certain because it concerned known missense variants (patient 6, p.(Arg190Gln) and patient 16, p.(Glu283Lys)) or a frameshift variant (patient 4, p.(Ser121Ilefs*21)). One variant was present in a mosaic state (patient 17, p.(Arg367*), see below).

After careful review, 2 of 32 patients were excluded from summary statistics. Patient 6 had a history of rubella encephalitis as a child, which may significantly influence the phenotype. Patient 17 had a very mild phenotype with normal development, borderline intellect, and a few tonic-clonic (TC) seizures at adult age, not consistent with a diagnosis of STXBP1-DEE. She carried a somatic mosaic pathogenic *STXBP1* variant in blood that was present in a heterozygous state in her daughter diagnosed with STXBP1-DEE. The following sections describe the features in the remaining cohort of 30 patients. The median age at last follow-up was 24 years (range 18–58 years).

### Epilepsy

All 30 patients had a history of epilepsy. The median age at seizure onset was 3.5 months (range: 1 day (with suspected seizures in utero) to 19 years), and 24 of 30 (80%) had seizure onset within the first year of life. At last follow-up, 5 of 30 (17%) patients had been seizure-free for 10–25 years, having become seizure-free at age 8 months to 11 years. Twenty-four of 30 (80%) patients had active epilepsy, with (several) seizures per day in 9 of 24 (38%) patients, weekly seizures in 10 of 24 (42%) patients, and monthly seizures in 4 of 24 (17%) patients. Patient 5 had a history of few TC seizures alone. In patient 31, possible very rare focal seizures were reported at last follow-up (not counted as having active epilepsy). Notably, 11 of 30 (37%) patients had prolonged seizure-free periods (range 1–16 years, median 3 years) in early childhood or adolescence with later seizure recurrence, and in 7 of them, ASMs could (temporarily) be withdrawn.

#### Evolution of Seizure Types

Patients had 1–3 different seizure types at onset. Focal seizures (n = 14), epileptic spasms (ES; n = 8), TC seizures (n = 4), and tonic seizures (n = 4) were most frequent. From infancy through adolescence, patients had 1–7 seizure types, most frequently including focal seizures (n = 21), tonic seizures (n = 19), TC seizures (n = 16), and ES (n = 11). Four patients had periods of status epilepticus (specified convulsive in 2). Adult patients still had 1–5 different seizure types, with TC seizures (n = 18), focal seizures (n = 16, often with impaired awareness and/or motor components), and tonic seizures (n = 11) as most frequent seizure types. Less frequent were atonic seizures (n = 4), myoclonic seizures (n = 3), and ES (n = 3, example in Video 1). Periods of status epilepticus were observed in 3 adult patients (specified convulsive in 1 and nonconvulsive in 1).

10.1212/200719_Video_1 Video 1 Video showing a cluster of violent epileptic spasms in a 19-year-old man with STXBP1-DEE.Download Supplementary Video 1 via http://dx.doi.org/10.1212/200719_Video_1

#### Evolution of EEG Pattern

The interictal EEG at epilepsy onset was normal in 4 of 21 (19%) patients. Focal or multifocal interictal epileptiform discharges (IEDs) were present in 10 of 21 (48%) and focal slowing in 5 of 21 (24%) patients. Five children had hypsarrhythmia and 2 with a burst suppression pattern. EEG in adults did not differ significantly from EEG during childhood through adolescence and showed background slowing (8/18 adults, 44%) with focal/multifocal IED (12/18, 67%) and/or focal slowing (5/18, 28%). Two patients always had a normal EEG, and in 2 additional patients, the EEG was normal at their most recent recording (at 17 and 54 years of age).

#### Response to Antiseizure Medication

The ASMs most frequently reported to be effective before adulthood were valproic acid (VPA, 13/26, 50%), benzodiazepines (clobazam [CLB]/clonazepam [CLN]/nitrazepam, 11/31, 35%), vigabatrin (10/16, 63%), lamotrigine (LTG, 7/16, 44%) as well as levetiracetam (LEV, 5/16, 31%), and phenobarbital (5/15, 33%). Ketogenic diet therapies (KDTs) reduced seizure frequency in 2 of 5 (40%) and adrenocorticotropic hormone in 4 of 6 (67%) patients. Adult patients with active epilepsy were (still) treated with 1–5 ASMs (mean: 3 ASMs) at last follow-up. The ASMs most frequently reported as effective in adulthood were LTG (6/14, 43%), VPA (6/14, 43%), and benzodiazepines (CLB/CLN, 5/14, 36%), followed by LEV (4/12, 33%) and topiramate (3/9, 33%). Vagal nerve stimulation was (partially) effective in 5 of 6 (83%) patients with vagus nerve stimulation and KDT in 1. (Denominator indicating the number of patients in whom antiseizure treatment was trialed.)

### Developmental Trajectory, Cognitive Outcome, and Behavioral Features

All patients had developmental delay that became apparent in the first 2 years of life and in 22 of 29 (76%) patients before the age of 1 year. In at least 7 patients, developmental delay preceded seizure onset. One or more episodes of regression of variable severity occurred in 16 of 27 (59%) patients. In 7 of 16 patients, episodes of regression correlated with seizure onset or increased seizure activity. Notably, 7 of 27 (26%) had loss of skills beyond early childhood (range 4–23 years), mainly affecting loss of verbal communication and walking. At last follow-up, 10 of 30 (33%) patients had profound or severe-profound ID, 16 of 30 (53%) severe ID, and 4 of 30 (13%) moderate or moderate-severe ID.

Eleven of 26 (42%) patients were diagnosed with autism spectrum disorder or showed autistic features. Stereotypies were noted in 14 of 30 (47%) patients mostly including hand stereotypies (n = 6), body rocking (n = 3), and previously described figure-of-eight head stereotypies (n = 2).^29^ Seventeen of 26 (65%) patients had behavioral or psychiatric problems, including aggressive behavior (n = 7), self-mutilation (n = 4), hyperactivity (n = 3), compulsive symptoms (n = 1), and episodes of psychosis or auditory hallucinations at adult age in 2 sisters. Seven adults received medication for behavioral/psychiatric problems at last follow-up.

### Clinical Neurologic Examination With Specific Focus on Movement Disorders and Extrapyramidal Features at Adult Age

#### Movement Disorders Including Gait Disturbance and Extrapyramidal Features Reported by the Referring Clinician

In 26 of 30 (87%) patients, movement disorders were reported by the referring clinicians (Table 1). The 4 patients in whom this was not described had severe spastic tetraparesis. Most frequently reported were tremor (17/30, 57%) and gait disturbances (present in 10/19 patients able to walk, 53%). Less frequent were hypomimia (8/30, 27%), bradykinesia (3/30, 10%), and rigidity (2/30, 7%), described as cogwheel rigidity in 1. Ataxia was not further specified in 8 patients. Patient 13 was effectively treated with propranolol for his tremor, and patient 1 was trialed on levodopa and rasagiline with uncertain effect.

**Table 1:**
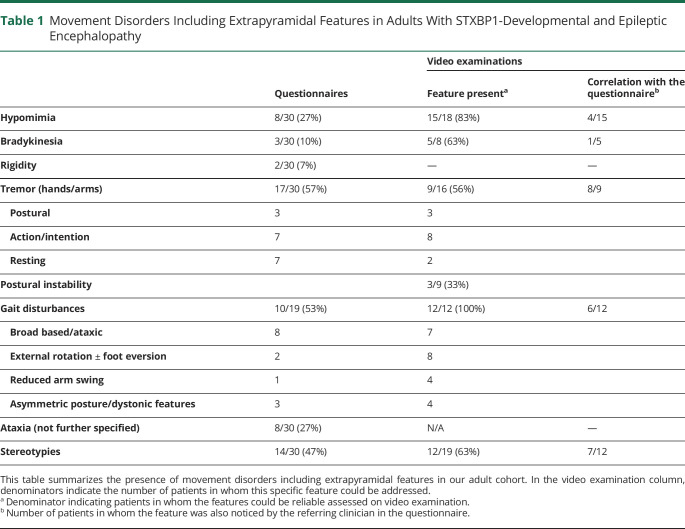
Movement Disorders Including Extrapyramidal Features in Adults With STXBP1-Developmental and Epileptic Encephalopathy

#### Movement Disorders Including Gait Disturbance and Extrapyramidal Features as Assessed by Video Examinations

For 19 of 30 (63%) patients, video recordings were available (Table 1 and eTable 3, links.lww.com/WNL/C93) including clinical examination capturing (some) items of the MDS-UPDRS motor subscale in 11 of 19 patients.

The most frequently observed features were hypomimia (15/18, 83%), bradykinesia (5/8, 63%), and different types of tremor (9/17, 56%) most frequently including intention/action tremor of the hands (n = 8). In 5 patients, the tremor was irregular or jerky, with clinically convincing myoclonic features in 2 of 5 patients. In all 12 patients able to walk (walking with limited assistance included), (mild) gait abnormalities were noted most frequently including postural abnormalities of the feet with external rotation with/without eversion (n = 8) and broad-based gait (n = 7). Stereotypies were observed in 12 of 19 (63%) patients, with figure-of-eight head stereotypies in 5 patients. Representative videos of gait abnormalities, tremor, and stereotypies are available in Videos 2 to 4. Videos of 2 patients showed choreatic dyskinesia (Video 5).

10.1212/200719_Video_2 Video 2 Video showing 4 adult patients with STXBP1-DEE with mild to pronounced gait abnormalities (1): a 21-year-old man with mildly broad-based gait with external rotation of the feet and pes planus; (2): a 19-year-old woman with more pronounced broad-based gait with external rotation and eversion of the feet while walking and pes planus; (3): a 20-year-old man with pronounced ataxic gait with external rotation of the feet; and (4): a 22-year-old woman with broad-based gait with asymmetric posturing and dystonia of the left arm and impression of hypertonia in the legsDownload Supplementary Video 2 via http://dx.doi.org/10.1212/200719_Video_2

10.1212/200719_Video_3 Video 3 Different tremor types in 3 adult patients with STXBP1-DEE: a jerky/irregular component is often present.Download Supplementary Video 3 via http://dx.doi.org/10.1212/200719_Video_3

10.1212/200719_Video_4 Video 4 Stereotypies including figure-of-eight head stereotypies in 3 adult patients with STXBP1-DEE.Download Supplementary Video 4 via http://dx.doi.org/10.1212/200719_Video_4

10.1212/200719_Video_5 Video 5 Dyskinesia including choreiform movements in 2 patients with STXBP1-DEE.Download Supplementary Video 5 via http://dx.doi.org/10.1212/200719_Video_5

#### Additional Neurologic Findings Reported by the Referring Clinician

Abnormal muscle tone was a frequent neurologic comorbidity and consisted of spasticity/hypertonia in 13 of 29 (45%) patients and (axial) hypotonia in 8 of 29 (28%) patients. Two patients had unilateral and bilateral foot drop suggesting possible (primary or secondary) involvement of the peripheral nervous system, although no nerve conduction studies were available. Furthermore, 2 patients had cortical visual impairment and 3 oral apraxia or dyspraxia.

### Neuroimaging

MRI reports were available for 29 of 30 (97%) patients. In 22 of 29 (76%) patients, brain MRI was normal including 7 of 9 patients in whom imaging was (also) performed at adult age. Four of 29 (14%) patients had some degree of cerebral atrophy. In 1 patient (patient 19), mild atrophy was reported at adult age but was not described in imaging reports at 4 and 13 years old. Four of 29 (14%) patients had other nonspecific imaging findings, including right parietal gyral asymmetry and FLAIR hyperintensities in patient 14, a small infarction in patient 23, small hippocampi with incomplete rotation in patient 24, and a suspected temporal myelination defect in patient 32.

### Non-neurologic Comorbidities

In 22 of 30 (73%) patients, non-neurologic comorbidities were reported. Fourteen of 30 (47%) patients reported gastrointestinal problems, including constipation (n = 6), problems with feeding/weight maintenance (n = 4), and gastroesophageal reflux disease (n = 2). Four of 30 (13%) patients received food/liquids through a percutaneous endoscopic gastrostomy tube (PEG-tube). Six of 30 (20%) patients were reported to have significant problems with sleep initiation or maintenance. Other recurrent non-neurologic comorbidities were (severe) scoliosis (n = 4), joint laxity (n = 3), and (mild) dysmorphic features (n = 3).

### Communication, Mobility, and Functional Independence in Adulthood

**Figure 1:**
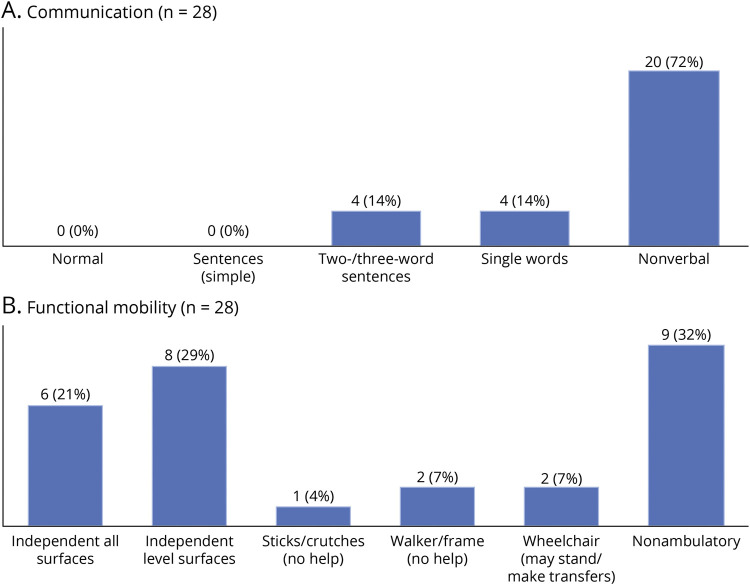
Functional Mobility and Level of Verbal Communication at Adult Age Bar charts showing the functional outcome at last follow-up concerning verbal communication (A) and mobility assessed with the Functional Mobility Scale (B).

**Figure 2:**
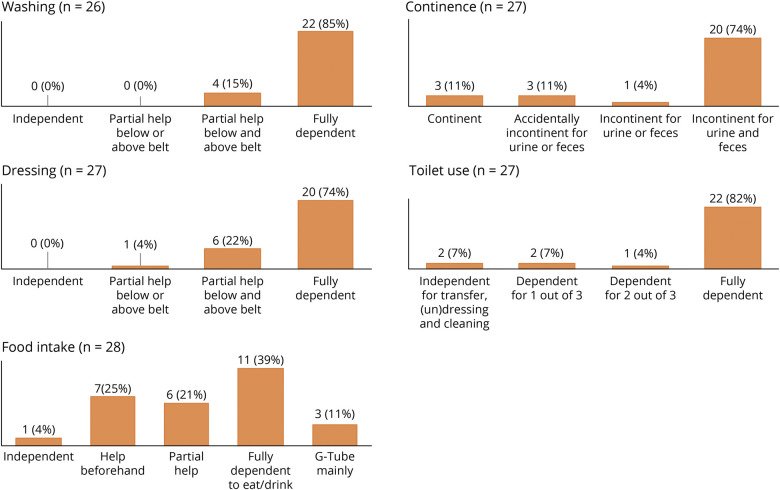
Level of Functional Independence at Adult Age Bar charts showing the functional outcome at last follow-up concerning activities of daily living assessed with the Katz scale.

Communication problems were present in all patients, with 20 of 28 (71%) individuals being nonverbal (Figure 1A). Motor disability was variable and ranged from walking (short distances) independently to completely nonambulatory (Figure 1B). All patients were largely dependent for activities of daily living, such as washing, dressing, toileting, and feeding (Figure 2). This need for constant care translated to a significant number of adult patients (14/27, 52%) living in residential care most of the time. Nine (33%) patients went to the daycare center, and 4 (15%) were at home predominantly.

### Death and Cause of Death

Two of 30 patients died after their last follow-up at the ages of 36 and 23 years. The reported cause of death was a postsurgical infection and sepsis, respectively.

### Genotypic and Phenotypic Correlations

No (statistically significant) correlation was found between variant type (missense vs other) and seizure outcome, cognitive outcome, presence of regression, and functional outcome concerning mobility and communication. Likewise, no correlation was found between seizure outcome (seizure-free vs not seizure-free) and cognitive outcome or functional outcome concerning mobility and communication. Of interest, there was a significant correlation between the age at seizure onset and the level of functional mobility at last follow-up, with the median age at seizure onset being lower in the group of patients who were wheelchair dependent (median: 25 days) compared with the group of ambulatory patients (median: 10 months; Mann-Whitney *U*, *p* = 0.004). This correlation was not seen regarding cognitive outcome, presence of regression, or the level of verbal communication (data available on request).

## Discussion

In this study, we analyzed the phenotypic features of STXBP1-DEE in adulthood to delineate the natural history of the disease. Our study cohort included 30 adult patients with disease-causing *STXBP1* variants including missense variants in about half of the patients, next to protein-truncating variants, indels, and splice-site variants.

Although epilepsy is not a mandatory feature of STXBP1-DEE, all patients included in our study had epilepsy, which was active at adult age in 80%. This may be related to the relatively small number of patients included in our study or due to referral bias because many patients were recruited through collaborating (child) neurologists with interest in epilepsy. Although seizures typically started in infancy or early childhood (before the age of 1 year in 80%), 1 patient had seizures starting at adult age only. Seizure-onset age beyond early childhood has been reported rarely in previous studies as well.^12^ Focal seizures and ES were the most frequent seizure types at onset, whereas in adulthood, TC seizures, focal seizures, and tonic seizures were most frequent. Notably, in 3 patients, ES were described in adulthood, which is uncommon because ES are epileptic events typical of infancy. Because these patients also had tonic seizures, we cannot exclude that in this context ES and tonic seizures may lie on a spectrum as the differentiation often relies on duration, with ES lasting less than 2 seconds and tonic seizures more than 2 seconds.^36^ However, ES in adults had the typical pattern of series of spasms seen in younger patients. As such, we propose that adolescents or adults with unsolved DEE with ongoing apparent ES deserve genetic scrutiny for *STXBP1*.

Although our cohort was limited because of size and potential recruitment bias, we stipulate that seizures in STXBP1-DEE may represent a lifelong burden and may continue to be drug resistant in adulthood. At last follow-up, 80% of this study cohort had active epilepsy with seizure frequencies ranging from multiple seizures a day to monthly seizures. In keeping with this, most adults still received multiple ASMs. Notably, more than a third experienced prolonged periods of seizure freedom in childhood or adolescence and in some patients to an extent that ASMs could be temporarily withdrawn. The underlying mechanisms for this phenomenon are unknown so far and represent an area in need of further investigation. Altogether, our results underscore the importance of continuous monitoring for seizures in adolescents and adults with STXBP1-DEE, including in patients who have been seizure-free for a prolonged period of time. A summary of key findings and recommendations for clinicians caring for (adult) patients with STXBP1-DEE can be found in Table 2.

**Table 2:**
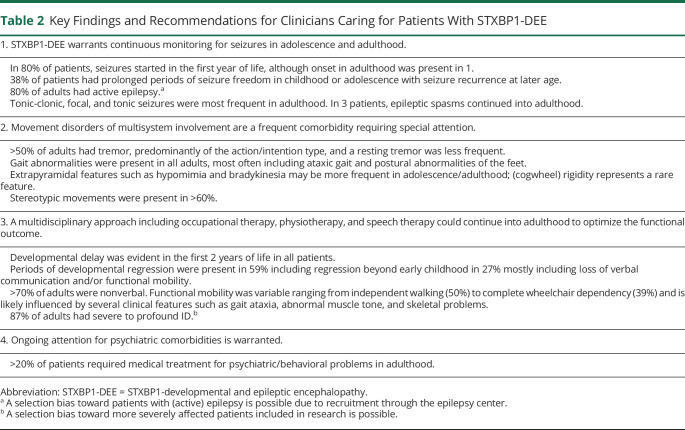
Key Findings and Recommendations for Clinicians Caring for Patients With STXBP1-DEE

Developmental delay is a core clinical feature of STXBP1-DEE and usually already evident in the first 2 years of life. Our study revealed episodes of regression in more than half of the patients, including loss of communicative or motor skills beyond early childhood in 26%, which is significantly more frequent than previously described.^12^ A correlation with seizure activity was not always present. None of the adults in this cohort had mild-moderate ID, although this was previously reported in a small proportion of patients. This may be partially explained by a selection bias toward patients with a more severe presentation that might favor inclusion in research studies. An alternative explanation could be an increased diversion from developmental expectations with age as was recently suggested in a study addressing developmental and behavioral characteristics in STXBP1-DEE.^37^ Problems with social interaction, including autistic features, often with additional behavioral problems were present in more than half of our cohort, and 23% received medical treatment for psychiatric/behavioral problems at last follow-up, indicating that behavioral problems still form a significant problem at adult age.

Through the use of semistructured video examinations including tasks from the motor part of the MDS-UPDRS, in addition to clinical questionnaires, we aimed to reduce interobserver variability and increase the possibility of identification of discriminatory findings. Structured video review resulted in a higher yield of movement abnormalities compared with data collection through questionnaires alone (Table 1). Gait abnormalities, including broad-based gait and postural abnormalities of the feet, were most frequent. Hypomimia was seen or reported in more than half of the patients but was often associated with a hypotonic facies, which would influence facial expression. Tremor, present in 56%, was mostly of the action/intention type. In the questionnaires, a resting tremor was more frequently reported than observed on video examinations, indicating that the momentary observations on video examination may not have captured all different tremor types. Nevertheless, central review of video material showed that tremors often had a jerky/irregular appearance with clear myoclonia in some. Of interest, and strengthening our finding, a recent study characterized the tremor in some STXBP1 patients as a tremor-like subcortical myoclonus.^38^ Altogether, these findings are not compatible with a pure extrapyramidal movement disorder and suggest multisystem involvement. Functional imaging studies including dopamine transporter imaging, although not straightforward in this patient population, could shed a light on the relative contribution of different systems involved. We further note that certain medications taken by some of this adult cohort, such as valproic acid and neuroleptic drugs, may also induce or worsen movement disorders such as tremor or bradykinesia. Of interest, 1 patient (patient 1) received treatment with levodopa (± rasagiline) after her neurologist noticed extrapyramidal features. On subsequent investigation by a different neurologist, these features were no longer observed. It is uncertain whether this represents a true effect of levodopa, but a trial of levodopa would be of interest in patients with STXBP1-DEE with disruptive extrapyramidal features.

All adult patients with STXBP1-DEE in our study had significant limitations in verbal communication, with the majority of patients (71%) being nonverbal. Functional mobility was more variable ranging from independent walking on all or level surfaces (50%) to being mostly or completely wheelchair dependent (39%). We note that some patients were able to walk short distances independently but used a wheelchair for longer distances. Mobility in STXBP1-DEE is likely influenced by several clinical features such as gait ataxia, (axial) hypotonia, spasticity, and dystonia and skeletal problems such as foot deformities and scoliosis. Of interest, we found a statistically significant correlation between age at seizure onset and level of functional mobility. Further investigation is warranted to look whether this can be replicated in larger study samples and whether this represents a causal relationship. Gastrointestinal problems were the most frequently reported non-neurologic comorbidities (47%), with 13% of patients requiring a PEG tube. All adult patients in our study were partially or completely dependent for most activities of daily living.

There are almost no data on life expectancy in STXBP1-DEE. Here, we report the oldest patient to date, a woman aged 58 years. Taking into account a previous report of a patient aged 56 years,^12^ we can conclude that patients with STXBP1-DEE can live up to their sixties at least. Two of 30 (7%) adult patients included in our study died in their 3rd and 4th decade. The cause of death in both patients was not related to seizures. However, our study recruited adults with STXBP1-DEE and consequently cannot assess early mortality in STXBP1-DEE. More systematic studies looking at the frequency and cause of death are warranted.

Although the early disease course of STXBP1-DEE suggests a primary problem in neurodevelopment, STXBP1 had also been implicated in neurodegeneration. First, both in vitro and in vivo studies have shown that STXBP1 is critical for neuronal survival and maintenance.^5,6^ Furthermore, a recent study revealed that STXBP1 controls the self-replicating aggregation of α-synuclein, a protein involved in various neurodegenerative diseases including Parkinson disease.^17^ By definition, neurodegenerative disorders deteriorate with age; hence, a study of older patients with STXBP1-DEE provides a unique opportunity to look for clinical signs of neurodegeneration. In this regard, the presence of developmental regression beyond early childhood (present in 26% in our cohort) was remarkable, especially because this was not always clearly related to flare-ups in seizure activity. However, these periods of regression occurred at only certain points in life, with plateauing rather than showing a protracted course. The mechanisms underlying this unusual phenomenon remain to be identified. Another argument favoring a role of neurodegeneration is the movement abnormalities we described, particularly the extrapyramidal features that seem to be more prevalent in older patients. We did not find convincing clinical evidence for neurodegeneration when comparing MRI reports from adult and childhood age, which showed possible mild progressive cerebral atrophy in only 1 of 7 patients with information available. Further studies using serial MRI are necessary to draw any further conclusions.

In this study, we focused on the clinical presentation of STXBP1-DEE in adulthood. We propose that this study could serve as a pilot study for future (prospective) natural history studies on STXBP1-DEE extending into adulthood.

Our study, however, has some limitations: the study design was cross-sectional with the inclusion of retrospective data and, as such, not ideal to evaluate disease evolution over time. Although the video examinations were of added value to the clinical questionnaires, we acknowledge they reflect momentary observations, and some clinical features of interest could not be reliably assessed on video.

Future natural history studies should preferably include longitudinal prospective data and use standardized scales and questionnaires adapted to the population studied to harmonize data collection. Repeated semistructured video examinations and serial brain MRI over time would be particularly interesting to further investigate the clinical correlates of a possible neurodegenerative component. The presence of ES in adulthood should be confirmed with simultaneous video-EEG and polygraphic recordings.

There is an unmet need for disease-modifying therapies for STXBP1-DEE because current therapeutic options are largely symptomatic and mainly directed at seizure control. Proceeding insights into the disease mechanisms underlying STXBP1-DEE are now paving the way for more targeted therapies that should tackle the whole gamut of comorbidities, including neurodevelopmental, movement disorders, behavioral and psychiatric and gastrointestinal problems, as well as seizures.^4^ Further studies confirming our findings suggestive of a slowly progressive disease course in at least some of the patients with STXBP1-DEE would provide a rationale to also study the effect of targeted therapy initiation in adolescence/adulthood. Understanding the natural history of STXBP1-DEE will be essential for the selection of relevant clinical outcome measures in future therapeutic trials.

## Glossary

ASM: antiseizure medication
CLB: clobazam
CLN: clonazepam
DEE: developmental and epileptic encephalopathy
ES: epileptic spasms
IEDs: interictal epileptiform discharges
KDT: ketogenic diet therapy
LEV: levetiracetam
LTG: lamotrigine
MDS-UPDRS: modified Unified Parkinson Disease Rating Scale
STXBP1: syntaxin-binding protein 1
TC: tonic-clonic
VPA: valproic acid
